# Zika Virus Exhibits Lineage-Specific Phenotypes in Cell Culture, in *Aedes aegypti* Mosquitoes, and in an Embryo Model

**DOI:** 10.3390/v9120383

**Published:** 2017-12-16

**Authors:** Katherine A. Willard, Leah Demakovsky, Blanka Tesla, Forrest T. Goodfellow, Steven L. Stice, Courtney C. Murdock, Melinda A. Brindley

**Affiliations:** 1 Department of Infectious Diseases, College of Veterinary Medicine, University of Georgia, Athens, GA 30602, USA; kwillard@uga.edu (K.A.W.); lrdemako@gmail.com (L.D.); btesla@uga.edu (B.T.); 2 Department of Animal and Dairy Science, Regenerative Bioscience Center, College of Agriculture and Environmental Science, University of Georgia, Athens, GA 30602, USA; forrestgoodfellow@gmail.com (F.T.G.); sstice@uga.edu (S.L.S.); 3 Department of Infectious Diseases, Odum School of Ecology, College of Veterinary Medicine, Center for Tropical Emerging and Global Diseases, Center for Ecology of Infectious Diseases, Center for Vaccines and Immunology, Riverbasin Center, University of Georgia, Athens, GA 30602, USA; cmurdock@uga.edu; 4 Department of Infectious Diseases, Department of Population Health, Center for Vaccines and Immunology, College of Veterinary Medicine, University of Georgia, Athens, GA 30602, USA

**Keywords:** zika virus, viral evolution, vector-borne disease, flavivirus, vector competence, *Aedes aegypti*

## Abstract

Zika virus (ZIKV) has quietly circulated in Africa and Southeast Asia for the past 65 years. However, the recent ZIKV epidemic in the Americas propelled this mosquito-borne virus to the forefront of flavivirus research. Based on historical evidence, ZIKV infections in Africa were sporadic and caused mild symptoms such as fever, skin rash, and general malaise. In contrast, recent Asian-lineage ZIKV infections in the Pacific Islands and the Americas are linked to birth defects and neurological disorders. The aim of this study is to compare replication, pathogenicity, and transmission efficiency of two historic and two contemporary ZIKV isolates in cell culture, the mosquito host, and an embryo model to determine if genetic variation between the African and Asian lineages results in phenotypic differences. While all tested isolates replicated at similar rates in Vero cells, the African isolates displayed more rapid viral replication in the mosquito C6/36 cell line, yet they exhibited poor infection rates in *Aedes aegypti* mosquitoes compared to the contemporary Asian-lineage isolates. All isolates could infect chicken embryos; however, infection with African isolates resulted in higher embryo mortality than infection with Asian-lineage isolates. These results suggest that genetic variation between ZIKV isolates can significantly alter experimental outcomes.

## 1. Introduction

Zika virus (ZIKV) is an emerging flavivirus that was first isolated from a sentinel Rhesus macaque in Uganda in 1947, yet was not identified as an important human pathogen until recently [1]. ZIKV is primarily transmitted through bites of *Aedes* spp. mosquitoes. However, the virus can also be contracted vertically and sexually [2,3,4]. Expansion of deforestation and agriculture has increased human exposure to sylvatic reservoirs. Additionally, globalization, intense urbanization, and adaptation to spread efficiently in *Ae. aegypti* have led to the explosive spread of many arboviruses, including Zika [5,6,7,8,9,10]. During geographic expansion, ZIKV evolved and differentiated into two, or possibly three, distinct lineages: African (ZIKV^AF^) and Asian (ZIKV^AS^) [11,12]; or African-1, African-2, and Asian/American [13,14]. There is also evidence of two distinct clades within the Asian-lineage: one containing isolates from the Pacific Islands and one containing isolates from the Americas [15]. Prior to the first large outbreak of ZIKV in Yap, Micronesia in 2007, there were few reported ZIKV infections worldwide [5]. These low case numbers could be attributed to low infection rates or to lack of reporting due to the generally mild and nonspecific symptoms of ZIKV infection, which include fever, joint pain, conjunctivitis, and skin rash [5]. However, retrospective analysis of samples taken at HIV and malaria clinics in Senegal and Nigeria indicate a 6.2% seroprevalence of ZIKV in these regions, suggesting active circulation during 1992–2016 [16]. These ZIKV infections may have been misdiagnosed, as ZIKV symptoms mimic those of other mosquito-borne diseases that circulate in the same areas, such as dengue fever [17] and chikungunya [18,19]. In 2015, an unusually high number of microcephaly cases were documented in Brazil in conjunction with the most extensive ZIKV outbreak to date [20]. Eventually, microcephaly was causally linked to congenital ZIKV^AS^ infection [21,22]. Recent work suggests that a S139N mutation in the prM protein of ZIKV^AS^ isolates associated with the American epidemic may have contributed to the increase in microcephaly cases [23]. In addition to microcephaly, ZIKV^AS^ infection is also associated with Guillain-Barré syndrome [24,25]. The drastic increase in birth defects and neurological symptoms associated with ZIKV infection may be due to differences in the pathogenicity between ZIKV^AS^ and ZIKV^AF^ or to differences in susceptibility and age of the exposed host population.

Numerous animal and cell culture studies on specific ZIKV isolates have been published since the American outbreak began [26,27,28,29,30,31]. Although these studies have produced valuable results, comparisons of the findings can be difficult due to variation among ZIKV isolates, experimental methods, and cell types used. For example, many studies use the original 1947 isolate MR766 as a model of ZIKV^AF^. However, MR766 was passaged in mouse brains and in vitro dozens of times after its isolation [1] and may no longer be representative of the ZIKV isolates that currently circulate in Africa [32]. The highly passaged laboratory African-lineage isolates lack a glycosylation site in the E protein, which is hypothesized to be a consequence of their in vitro passage history [12,33,34]. Glycosylation deletions in other flaviviruses are linked to specific phenotypic changes among viral lineages [35]. To circumvent this limitation, many contemporary ZIKV studies use ZIKV^AS^ isolates. For instance, isolates from the 2013 French Polynesian outbreak were frequently used until clinical isolates from the current epidemic in Latin America and the Caribbean became available. While ZIKV^AS^ isolates are highly conserved, studies have found that different isolates behave distinctly in a number of assays and model systems. The inconsistent use of ZIKV isolates raises the possibility that there may be additional lineage or isolate-specific phenotypic differences that have not yet been observed or well-defined.

Recently published data show lineage-specific differences in ZIKV dynamics in specialized cell types, including neural cells [31,32,36], placental trophoblasts [30], and dendritic cells [27]. For example, data show that ZIKV^AF^ infects significantly more neural stem cells and astrocytes and produces significantly higher viral titers than ZIKV^AS^ [32]. Additional studies have compared ZIKV isolates in a specific model system, such as mosquitoes [37,38,39], mice [40,41], or non-human primates [34,42]. While these results offer valuable insight into ZIKV outcomes in specific tissues or a particular model system, lineage-specific ZIKV infection dynamics have not been systematically and directly compared among distinct ZIKV isolates in cell culture, developing embryos, and mosquitoes.

To develop lineage-specific characterizations of ZIKV infection, we compared the in vitro growth rates of two ZIKV^AS^ isolates (SPH and Mex 1-44) and two ZIKV^AF^ isolates (IbH and MR766) in both mammalian and mosquito cells and tested their abilities to overcome the human interferon β-1A (IFN-β) immune response. We also offered *Ae. aegypti* mosquitoes infectious blood meals to determine the probability of becoming infected, establishing a disseminating infection, becoming infectious, the midgut escape efficiency, and salivary gland infection efficiency of each isolate. Finally, we inoculated two-day-old chicken embryos and observed embryo mortality following infection [43,44]. The results of these comparison experiments demonstrate that isolates from different lineages can display distinct phenotypes in vitro, in the mosquito vector, and in an embryo model.

## 2. Materials and Methods

### 2.1. Cell Lines

Vero cells were maintained in Dulbecco’s Modification of Eagle’s Medium (DMEM) with 5% fetal bovine serum (FBS) at 37 **°**C, 5% CO_2_. C6/36 *Ae. albopictus* cells (ATCC CRL-1660) were maintained in L-15 Leibovitz Medium with l-glutamine and 10% FBS at 28 **°**C.

### 2.2. Zika Virus Isolates

A summary of the ZIKV isolates used in this study and their corresponding passage histories can be found in Table 1. Asian-lineage SPH was isolated from a Brazilian clinical sample in 2015, passaged in Vero cells, and obtained from the Oswaldo Cruz Foundation (FIOCRUZ). Asian-lineage Mex 1-44 was isolated from an infected *Ae. aegypti* mosquito in Chiapas, Mexico in 2015, passaged in Vero cells, and was then obtained from the University of Texas Medical Branch. Both the African-lineage isolates MR766 and IbH were obtained from the American Type Culture Collection (ATCC-catalog numbers VR-1838™ and VR-1829™, respectively) and passaged in Vero cells in the laboratory. Prior to experimentation, all ZIKV isolates tested negative for Mycoplasma contamination (MycoSensor PCR Assay Kit-Agilent, West Cedar Creek, TX, USA), were titered, and were normalized to 10^5^ tissue culture infectious dose 50 (TCID_50_) units/mL on Vero cells.

### 2.3. Determination of Viral Titers

ZIKV titers were determined using the Spearman-Karber TCID_50_ method [46]. ZIKV plaque morphologies and mosquito blood meal titers were determined using plaque assays. Briefly, Vero cells were plated and incubated until monolayer formation. The cells were then infected with either 100 plaque forming unites (PFU) (plaque morphologies) or 10-fold serial dilutions (blood meal titers) of ZIKV and incubated for 2–4 h. After infection, the supernatant was removed and the cells were overlaid with a 1.5% UltraPure low melting point agarose (Invitrogen, Carlsbad, CA, USA)/DMEM mixture. The cells incubated until plaques were visible (4 days following infection) and were then fixed with 4% formaldehyde in phosphate buffered saline (PBS) and stained with crystal violet.

### 2.4. Viral Replication Curves

Vero and C6/36 cells were seeded in 12-well plates at a density of 2.5 × 10^5^ and 5.0 × 10^5^ cells/well, respectively. Once cells adhered to the plate (2–4 h), they were infected with the four ZIKV isolates at multiplicity of infection (MOI) 0.1 and MOI 1. After 2 h, the infectious supernatant was removed and 1 mL of fresh media was added to each well. At the indicated time points, 500 μL of supernatant was collected and replaced with the same volume of fresh media. For both Vero and C6/36 cells, time point 0 was sampled immediately after the infectious supernatant was exchanged for fresh media. All samples were stored at −80 **°**C before viral titers were determined using TCID_50_ in Vero cells.

### 2.5. Cell Metabolic Activity/Viability

Vero and C6/36 cells were seeded in 96-well plates at a density of 3.75 × 10^4^ and 5.75 × 10^4^ cells/well respectively and incubated for 2–4 h. The cells were then infected with the four ZIKV isolates at MOI 0.1 and MOI 1. After an additional 2 h, the infectious media was removed and replaced with fresh media. Every 24 h, the media was exchanged to mimic the viral replication experiment described above. Cellular metabolism (ATP abundance), which corresponds to cell viability, was assessed at the indicated time points using CellTiter-Glo (Promega, Madison, WI, USA) and a GloMax plate reader (Promega) as per manufacturer’s instructions. Infected cell viability was compared to uninfected controls.

### 2.6. Human Interferon-Beta 1A Response

The effect of human IFN-β on ZIKV replication was measured as previously described for West Nile virus, with slight modifications [47]. Vero cells were seeded in a 12-well plate at a density of 3.5 × 10^5^ cells/well and incubated for 3–5 h. Cells were then treated with 10,000 U/mL of human interferon beta 1A (Millipore-IF014, Temecula, CA, USA) and incubated for 24 h. Untreated cells served as controls. The cells were infected with the four ZIKV isolates (MOI 0.1), and the infectious IFN-β-containing media was removed after 1 h and was replaced with fresh media containing an additional 10,000 U/mL IFN-β. Supernatant samples were collected at the indicated time points and were replaced by an equal volume of fresh media. Viral titers were determined using TCID_50_ in Vero cells. Results are reported as the Log_10_ ratio of viral titers from treated cells to viral titers from untreated cells.

### 2.7. Intracellular STAT2 Abundance

Vero cells were seeded in 12-well plates at a density of 3.5 × 10^5^ cells/well and incubated for approximately 30 h. After incubation, the cells were infected with the four ZIKV isolates (MOI 0.1). The infectious media was removed after 1 h and replaced with 1 mL of fresh media. At the indicated time points, the supernatant was removed, the cells were washed twice in 1 mL of cold PBS, and the cells were lysed in 250 µL of M2 lysis buffer (5% 1 M Tris HCl *v*/*v*, 3% 5 M NaCl *v*/*v*, 0.2% 0.5 M EDTA *v*/*v*, 1% Triton X-100 *v*/*v*) for 10 min at 4 **°**C. The lysates were transferred to 1.5 mL tubes and centrifuged at 17,000× *g* for 5 min at 4 **°**C, and 200 µL of supernatants were transferred to fresh tubes. Aliquots (40 µL) of the cellular lysates were mixed with 10 µL of Urea SDS with dithiothreitol (DTT) and incubated at 56 **°**C for 10 min, then 20 µL of the denatured lysates were run on 10% Tris-glycine gels (Invitrogen, Carlsbad, CA, USA). After proteins were transferred to polyvinylidene fluoride (PVDF) membranes (GE Healthcare, Little Chalfont, UK) and blocked in 10% nonfat milk/PBS-T, signal transducer and activator of transcription 2 (STAT2) was probed with anti-Stat2 (B-3) primary antibody (Santa Cruz Biotech, Dallas, TX, USA) at a 1:100 dilution and anti-mouse horseradish peroxidase (HRP) secondary antibody (Jackson Immuno Research, West Grove, PA, USA) at a 1:10,000 dilution. The immunoblots were imaged with SuperSignal West Femto Substrate (Thermo, Rockford, IL, USA) and a BioRad XRS+ ChemiDoc (Hercules, CA, USA). Results are reported as the band density of infected cells compared to that of uninfected cells across three independent trials. Two gels were used due to the number of samples. However, the same uninfected control was loaded onto both gels, therefore all samples are compared to the same control.

### 2.8. Ae. aegypti Rearing and Colony Maintenance

*Ae. aegypti* eggs were collected in Chiapas, Mexico by Dr. Américo David Rodríguez Ramírez of the Instituto National de Salud Pública and sent to our ACL2 insectary at the University of Georgia. The F1 adults were provided water and 10% sucrose (*w*/*v*) ad libitum and maintained at 27 ± 1 **°**C and 80% relative humidity on a 12 h:12 h dark:light cycle. Membrane adaptation of the F1 adults was achieved using O-positive human blood from a male donor (Interstate Blood Bank), which was provided in water-jacketed glass membrane feeders covered with a porcine sausage casing membrane. F2–F3 females were used for experimental feedings.

Mosquitoes emerged and were allowed to mate in the ACL2 insectary. Three-day-old females were sorted into 16 ounce cups (100 individuals/cup) and transferred to the ACL3 laboratory, where they were housed at 27 ± 0.5 **°**C and 80% relative humidity on a 12 h:12 h dark:light cycle for 48 h before the infectious feed. Mosquitoes were provided water ad libitum for the first 36 h after transfer, at which point the water was removed. After the blood meal, mosquitoes were once again given ad libitum access to 10% sucrose and water for 12–13 days. Sucrose was removed 48 h prior to forced salivation, though the mosquitoes were provided with ad libitum access to water. All water and sucrose pads were replenished daily.

### 2.9. ZIKV Infection in Ae. aegypti Mosquitoes

Washed red blood cells (RBCs) were freshly prepared before infectious blood feeds. In brief, O-positive human blood was washed twice in Roswell Park Memorial Institute 1640 (RPMI) media, pelleted at 1200× *g* for 10 min, then suspended in 33% (*v*/*v*) DMEM, 20% (*v*/*v*) FBS, 1% (*w*/*v*) sucrose, and 5 mM adenosine triphosphate (ATP). This RBC mixture was combined 1:1 with each ZIKV isolate to achieve a final viral titer of 10^6^ PFU/mL. The blood was immediately transferred to the ACL3 laboratory and was warmed to 37 **°**C for 15 min prior to the 30 min blood feed. Each cup of 100 mosquitoes was provided one glass feeder containing 2 mL of infectious blood. The blood meal was maintained at 37 **°**C throughout the feed with a circulating water pump. To record the infectious blood meal titer at the time of the feed, blood samples were collected immediately before and after the feed and titered via plaque assay. After feeding, non-engorged mosquitoes were removed and euthanized.

Mosquitoes were harvested 14 to 15 days post-infection (dpi). Mosquitoes were briefly anesthetized at −20 **°**C, and legs and wings were removed on a chill table (Bioquip, Rancho Dominiguez, CA, USA), allowing for insertion of the proboscises into pipette tips for saliva collection. Each tip contained 35 μL of a freshly prepared mixture of FBS, 3 mM ATP, and red food coloring (used to help determine if a mosquito imbibed the FBS media), which was warmed to 37 **°**C for 10 min prior to use. The immobilized mosquitoes were warmed to 35 **°**C for 45 min to collect saliva samples, after which the heads and bodies were dissected and collected. All samples were suspended in 600 µL of DMEM with 10% antibiotic/antimycotic, returned to the BSL2 laboratory, and stored at −80 **°**C. Head and body samples were homogenized using stainless steel beads (Qiagen TissueLyser II, Hilden, Germany) then centrifuged at 17,000× *g* for 5 min at 4 **°**C. Vero cells were infected with 250 μL of the supernatant from each sample; all samples with visible cytopathic effect (CPE) 5 dpi were considered positive for ZIKV.

### 2.10. ZIKV Inoculations in Chicken Embryos

All studies were conducted in accordance with NIH and University of Georgia, Athens (UGA) guidelines for embryonated chicken eggs. All virus work was approved by the UGA’s Institutional Biosafety Committee (2015-0022, approved 26 March 2015).

ZIKV infection of chicken embryos was conducted as previously described [44]. Embryonated broiler chicken eggs were obtained from the University of Georgia Poultry Science Farm and set in a Brinsea Ova-Easy Advance Incubator 2.5 days prior to infection. On the day of infection, eggshells were sanitized with 70% ethanol and reinforced with tape. Next, 1–3 mL of albumin was removed from each egg with a needle and syringe, and the hole was re-sealed with hot glue. Before infection, each viral isolate was diluted to inject a given number of viral particles per egg. The diluted stock was titered using TCID_50_ to determine the final virus concentration. A small hole was then cut into the top of each egg, and approximately 2 µL of virus or sham mixture (DMEM/5% FBS and Vero cell supernatant) was injected into each embryo using a microinjector; eighteen eggs were used per condition (sham or virus). A solution of penicillin and streptomycin was added, and the egg was re-sealed with a glass coverslip and hot glue. Embryo viability was periodically monitored with a bright light to visually detect a heartbeat and/or movement. Dead embryos were dissected, weighed, and collected to titer. Embryos that survived the full time course of the experiment (15 dpi) were culled before hatching, and their organs (brain, eyes, heart, liver, crop, and intestines) were collected to titer. Before titering, the total sample volume was brought to 1.5 mL with DMEM/antibiotic and antimycotic, and the tissue was macerated in a Qiagen TissueLyserII with a steel bead. The samples were centrifuged and the supernatant was titered via TCID_50_.

### 2.11. Statistical Analysis

ZIKV growth curves, cell viability, human IFN-β, and STAT2 results were all analyzed with the Student’s unpaired *t*-test (Graphpad QuickCalcs, https://www.graphpad.com/quickcalcs/, GraphPad Software, La Jolla, CA, USA). We used mixed effects generalized linear models (binomial distribution, random factor replicate) to estimate the effect of ZIKV strain (fixed factor) on the probability of a mosquito becoming infected, disseminating infection, and becoming infectious based on presence/absence of ZIKV in the bodies, heads, and saliva, respectively. Because the midgut and salivary gland barriers represent significant bottlenecks for arboviral infection, we used the same modeling framework to assess the probability of ZIKV escaping the midgut (of those with positive bodies, what is the probability of dissemination) and invading the salivary glands (of those with positive heads, what is the probability of becoming infectious). All alpha values associated with interpreting multiple comparisons were adjusted using Tukey honest significant difference (HSD) significance tests, and model performance/fit was assessed through checking model residuals Statistical Package for the Social Sciences (SPSS), IbM 24.0, Armonk, NY, USA). Chicken embryo survival values were generated and analyzed with Graphpad Prism (version 7, GraphPad Software, La Jolla, CA, USA). *p*-values are as follows: * *p* < 0.05, ** *p* < 0.01, *** *p* < 0.001, NS—not significant.

## 3. Results

### 3.1. ZIKV Plaque Morphology Varies Based on Lineage

**Figure 1:**
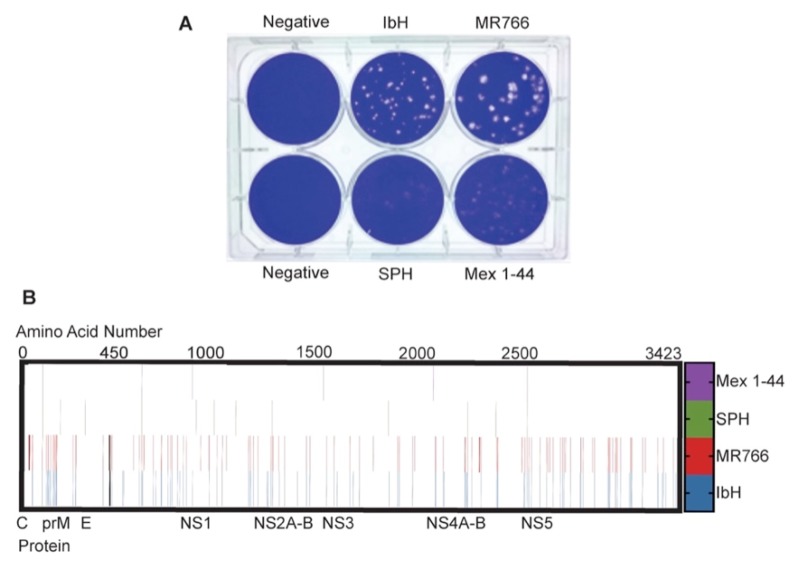
Genetic and phenotypic differences of Zika virus (ZIKV) lineages: (**A**) Plaque morphologies of the ZIKV isolates used in this study when stained at 4 days post-infection (dpi). Vero cells were infected with 100 plaque forming units (PFU) of each isolate; (**B**) Changes from the ZIKV consensus sequence across the translated ZIKV genome. Each line represents an amino acid change from the standard consensus sequence of ZIKV (PRVABC59-Genbank accession number KX087101.3). Black lines represent the E protein glycosylation site deletions in ZIKV^AF^.

While performing routine plaque assays with ZIKV, we noted differences in plaque morphologies when Vero cells were infected with ZIKV^AS^ versus ZIKV^AF^ isolates (Figure 1A). Under our plaque assay conditions, Asian-lineage isolates produced hazy, larger, and less-defined plaques, whereas African-lineage isolates produced a mixture of large and small plaques with very clear borders when stained at 4 dpi. SPH plaques were very diffuse, making them difficult to accurately count. Because ZIKV^AS^ did not produce clear plaques, viral titers for all ZIKV isolates were determined by TCID_50_ in Vero cells, as CPE was clearly evident with all strains. Protein sequence alignment of the isolates used in this study reveal that the ZIKV^AS^ isolates have approximately 3.5% amino acid variation from the ZIKV^AF^ isolates (Figure 1B), which corresponds with previously reported results [12]. This amino acid variation includes unique deletions in the E protein glycosylation sites of ZIKV^AF^ isolates IbH and MR766. Therefore, this genetic variation may contribute to the differential plaque morphologies.

### 3.2. ZIKV Lineage Impacts Vero Cell Viability

**Figure 2:**
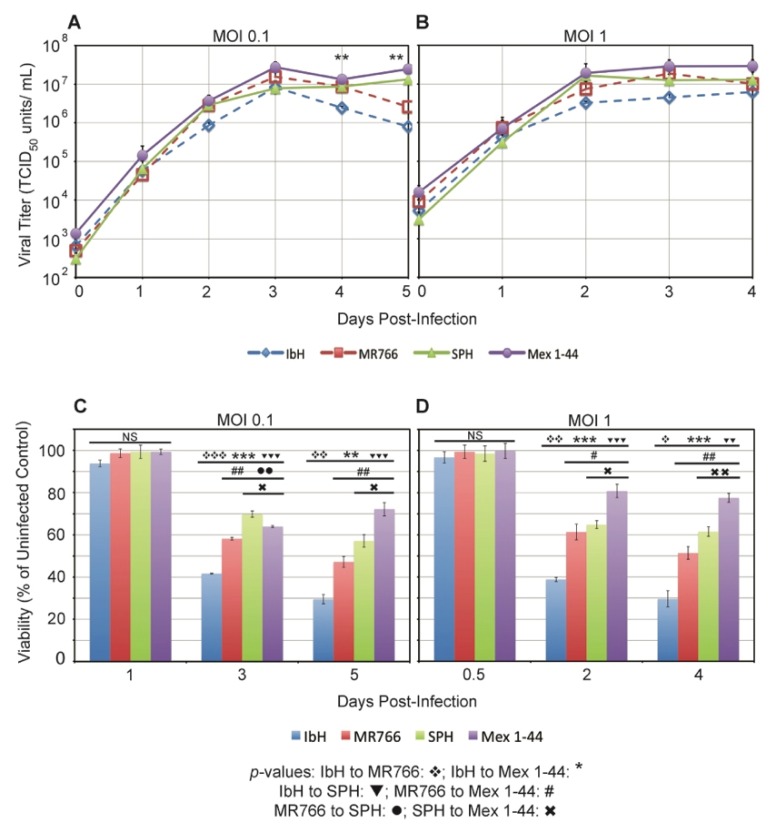
ZIKV replication and impact on viability in Vero cells. ZIKV replication rate in Vero cells at: (**A**) multiplicity of infection (MOI) 0.1; and (**B**) MOI 1. Data represent the average titer ± standard error of the mean (SEM) from three independent experiments; *p*-values were calculated using a student’s *t*-test with the *ln*-converted titers (** p < 0.1). Average viability of Vero cells infected with: (**C**) MOI 0.1 of ZIKV; and (**D**) MOI 1 of ZIKV. Data represent the average ± SEM percent of viable cells in infected wells compared to uninfected Vero cells from three independent trials; * *p* < 0.05, ** *p* < 0.01, and *** *p* < 0.001 by student’s *t*-test. NS: not significant.

To characterize the replication rates of ZIKV^AF^ and ZIKV^AS^ in mammalian cells, we infected Vero cells and monitored viral production and subsequent cell viability. Viral replication kinetics in Vero cells infected with MOI 0.1 were very similar among the four ZIKV isolates until three days post-infection (dpi) (Figure 2A). After 3 dpi, the ZIKV^AF^ titers began to drop, whereas the ZIKV^AS^ titers plateaued at a high level. Similar viral growth patterns were observed among ZIKV isolates in Vero cells infected at MOI 1, though viral replication occurred more quickly with peak titers occurring closer to 2 dpi (Figure 2B). Overall, these results indicate that there are minimal lineage-specific differences in ZIKV replication patterns in an interferon-deficient mammalian cell line [48,49].

Late during viral replication, ZIKV^AF^ titers began to fall, which correlated with obvious cytopathic effect (CPE). To monitor viability of infected cells, Vero cells were infected with the four ZIKV isolates and metabolic activity was measured over time. Based on the corresponding growth curves, we chose time points when viral growth was exponential, when it plateaued, and when titers began to drop. Vero cell viability differed by lineage and by isolate, with ZIKV^AF^ isolate IbH being the most cytopathic at both MOIs (Figure 2C,D). Interestingly, the CPE pattern closely followed that of viral titer; the most cytopathic isolates had the lowest viral titers over time. Although cytopathicity varied by isolate, the values also segregated by lineage, with the ZIKV^AF^ isolates overall being significantly more cytopathic than ZIKV^AS^ isolates. Therefore, ZIKV lineage impacts Vero cell viability.

### 3.3. ZIKV Response to Human IFN-β Treatment Varies by Isolate

**Figure 3:**
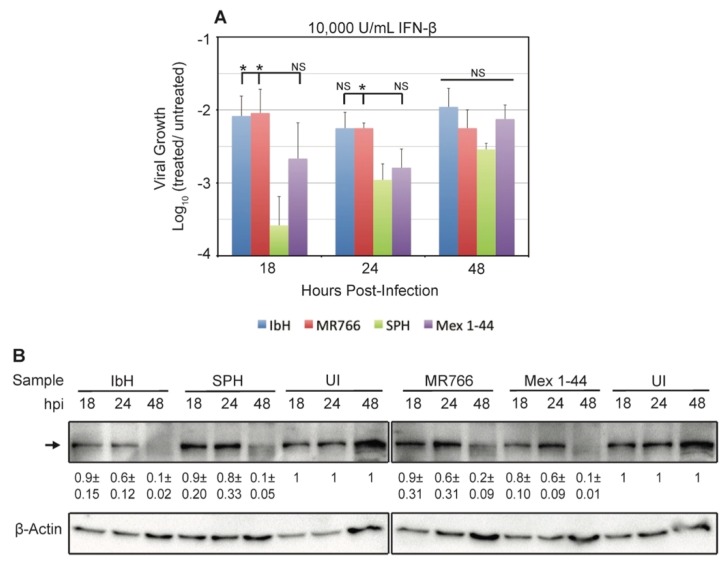
ZIKV response to interferon beta (IFN-β) treatment. (**A**) Average ZIKV growth in Vero cells treated with 10,000 U/mL of human IFN-β. Viral growth was measured via tissue culture infectious dose 50 (TCID_50_). The data are displayed as the Log_10_ ratio of viral titers from interferon-treated cells to viral titers from untreated controls three independent trials. Data represents the average ± SEM. Significance is shown in relation to SPH, * *p* < 0.05; (**B**) Immunoblot of intracellular signal transducer and activator of transcription 2 (STAT2) in ZIKV-infected Vero cells. The image is a representative from three independent trials. STAT2 levels were quantified by comparing the STAT2 levels at each time point with the uninfected control; the average ratios ± SEM are provided under the representative bands. The uninfected control is the same sample in both panels. All interactions between isolates within a time point are NS by the student’s *t*-test. The increase in UI band density over time is attributed to the increase in cell density at later time points. The increase in the β-Actin loading control band density over time indicates cell replication, meaning the decreases in STAT2 levels in infected cells are not due to cell death.

Interferon-β (IFN-β) is an important component of the innate immune response against ZIKV [28,40,50,51,52,53,54] and other flaviviruses [55,56]. Vero cells have a genomic deletion that eliminates their interferon gene cluster, so they cannot produce IFN-β in response to viral infection [48,49]. However, they are still able to respond to exogenous interferon through STAT2 signaling, making them a useful tool to determine if ZIKV^AF^ and ZIKV^AS^ are differentially affected by IFN-β. We found that all ZIKV isolates were affected by treatment with human IFN-β; all viral titers decreased when cells were treated with 10,000 U/mL of IFN-β compared to untreated cells (Figure 3A). The largest titer decreases occurred early during infection, and titers rose or remained constant as the effect of IFN-β diminished over time. Isolate SPH was significantly more affected by IFN-β treatment than the ZIKV^AF^ isolates, but its viral production was never significantly different from Mex 1-44.

To determine a mechanism for these differential responses to interferon, we measured the amount of STAT2 in ZIKV-infected Vero cells. A previous study demonstrated that ZIKV NS5 degrades host cell STAT2, which antagonizes downstream activation of interferon genes. The same study also found that the level of STAT2 degradation does not depend on ZIKV lineage [28]. Because our ZIKV^AF^ and ZIKV^AS^ isolates have many amino acid differences in NS5 (Figure 1B), we tested if STAT2 degradation could explain our IFN-β results. Our results are comparable to those of the previous study, with similar degradation of STAT2 over time in cells infected with the different ZIKV isolates (Figure 3B). Therefore, the differential responses to IFN-β are not a result of isolate-specific STAT2 degradation in host cells.

### 3.4. ZIKV Lineage Impacts Growth Kinetics in C6/36 Mosquito Cells

**Figure 4:**
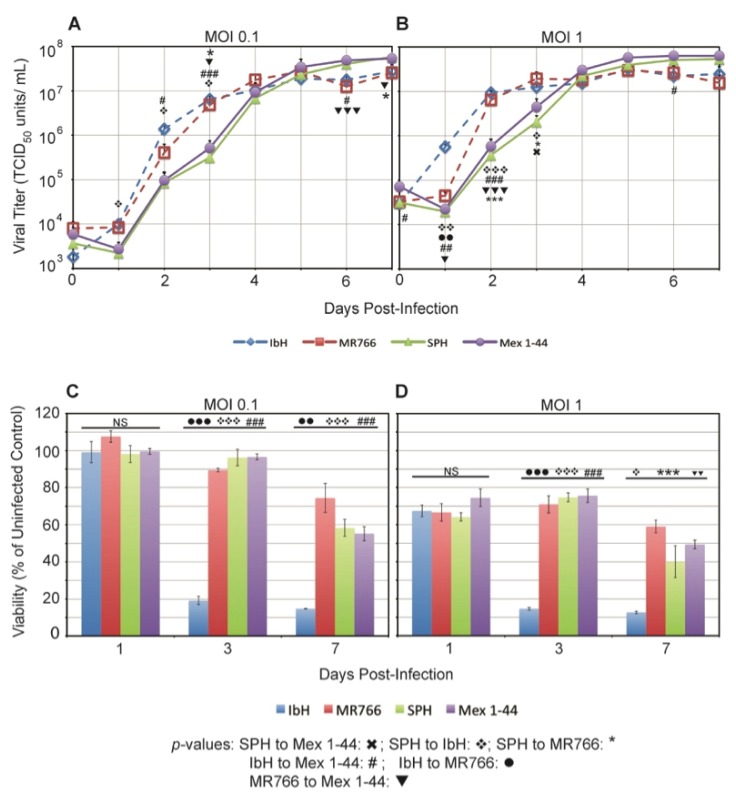
ZIKV replication in C6/36 cells. ZIKV average growth in C6/36 cells at: (**A**) MOI 0.1 and (**B**) MOI 1. Data represent the average ± SEM titer from three independent experiments; *p*-values were calculated using a student’s *t*-test with the *ln*-converted titers. Average viability of C6/36 cells infected with: (**C**) MOI 0.1 of ZIKV; and (**D**) MOI 1 of ZIKV. * *p* < 0.05, ** *p* < 0.01, and *** *p* < 0.001.

We infected C6/36 *Ae. albopictus* cells with ZIKV^AF^ and ZIKV^AS^ and compared the viral replication rates of the four ZIKV isolates. In cells infected with both MOI 0.1 and MOI 1, African-lineage isolates produced infectious particles at a faster rate than their Asian-lineage counterparts during the first 3 dpi (Figure 4A,B). However, by 6 dpi, ZIKV^AS^ isolates reached higher viral titers than ZIKV^AF^. The pattern of ZIKV growth was similar for both MOI 0.1 and MOI 1, though replication for all isolates occurred more rapidly at MOI 1 (Figure 4B). Unlike Vero cells, in which all isolates replicated at similar rates, ZIKV replication in C6/36 cells segregated by lineage.

We did not observe any obvious CPE in C6/36 cells despite the high viral titers produced from ZIKV infection. However, there were differences in metabolic activity/viability when cells were infected with the different ZIKV isolates. Infection with ZIKV^AF^ isolate IbH resulted in significantly lower viability than infection with the other isolates at both MOI 0.1 (Figure 4C) and MOI 1 (Figure 4D). Interestingly, this metabolic activity/viability did not segregate by lineage as it did in the Vero cells. As expected, viability was lower overall in cells infected with MOI 1 compared to MOI 0.1.

### 3.5. Vector Competence of Aedes aegypti Depends on ZIKV Lineage

**Figure 5:**
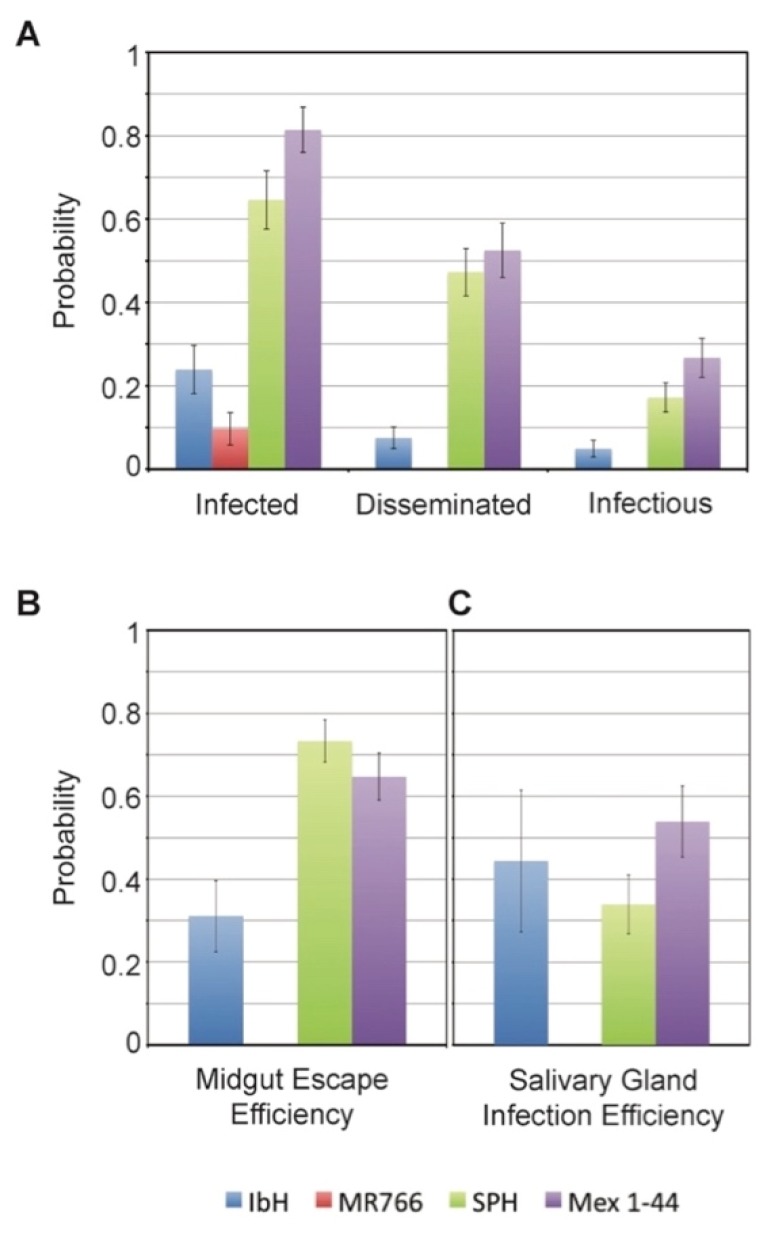
ZIKV dynamics in *Aedes aegypti* mosquitoes. *Ae. aegypti* mosquitoes were fed an infectious blood meal containing one the four ZIKV isolates (approximately 1.5 × 10^6^ TCID_50_ U/mL) and were maintained for 14–15 days before processing. (**A**) We determined the probability of mosquitoes becoming infected (ZIKV positive bodies compared to total number of engorged), having disseminated infections (ZIKV positive heads compared to total number engorged), and becoming potentially infectious (ZIKV positive saliva samples compared to total number engorged); (**B**) To examine the efficiency of the different ZIKV isolates to disseminate throughout the mosquito, we also compared the midgut escape efficiency (ZIKV positive heads compared to ZIKV positive bodies); (**C**) To compare the ability of the isolates to infect the salivary gland, we determined the salivary gland infection efficiency (ZIKV positive saliva compared to ZIKV positive heads). Statistics for the data are shown in Table 2.

The vast majority of ZIKV transmission occurs through the bite of an infected *Aedes* mosquito [2]. During mosquito infection, the virus must first establish infection in the mosquito midgut and disseminate throughout the mosquito before it can enter the saliva and potentially spread to a new host. We found significant effects of ZIKV lineage on the probability of mosquitoes becoming infected, disseminating infection, becoming infectious, and the efficiency of escaping the midgut (Figure 5A,B, Table 2). Overall, mosquitoes have a significantly higher probability of being infected, disseminating infection, and becoming infectious with ZIKV^AS^ isolates as compared to those exposed to ZIKV^AF^ isolates (Figure 5A, Table 2 and Table 3). ZIKV^AS^ isolates were also more efficient in escaping the mosquito midgut relative to the ZIKV^AF^ isolates (Figure 5B, Table 2). After escaping the midgut barrier, we did not observe any significant differences among ZIKV isolates in their efficiency in invading the salivary glands (Figure 5C, Table 2).

### 3.6. ZIKV Lineages Cause Differential Mortalities in Chicken Embryos

**Figure 6:**
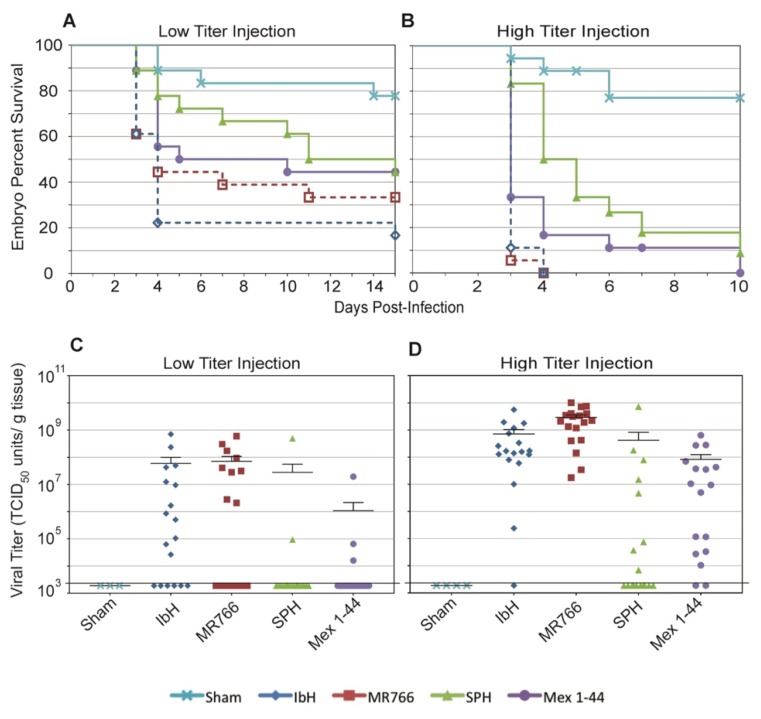
Chicken embryo response to ZIKV infection. Chicken embryo percent survival after: (**A**) low titer ZIKV infection; and (**B**) high titer ZIKV infection. *n* = 18 for each condition at day zero. Any eggs contaminated with bacteria or fungus were censored from the study. Statistics for these data are listed in Table 6. ZIKV titers of dissected embryonic tissues infected with: (**C**) low titer ZIKV; and (**D**) high titer ZIKV. Each symbol represents one embryo. Mean and SEM are shown for each condition. Percent survival and titers for sham-injected eggs are also shown. The value 1.89 × 10^3^ TCID_50_ units/gram of tissue (indicated by a bold horizontal line on the graphs) represents the lowest limit of detection of the TCID_50_ assay; therefore, data points at this level are considered negative for ZIKV.

Embryo models of ZIKV infection are necessary to understand ZIKV^AS^-associated birth defects; however, many mammalian embryo models are costly and difficult to examine longitudinally. Thus, chicken embryos are a useful model to study embryonic ZIKV infection as they are relatively inexpensive, and individual embryo outcomes can easily be monitored over time [44,57]. When we employed this model, we found that chicken embryos infected with ZIKV^AS^ had a higher probability of survival than those infected with ZIKV^AF^, especially at early times following infection. This result is consistent when embryos were infected with both low (Figure 6A) and high titers (Figure 6B) of ZIKV.

Our intention was to infect with either 4 or 40 Zika viral particles per egg. However, the actual number of ZIKV^AS^ particles injected was higher than the number of ZIKV^AF^ particles injected (Table 4). Despite this dosage difference, the ZIKV^AF^ isolates infected more eggs (Figure 6C,D), resulted in tissues with higher average viral titer (Table 5), and produced higher embryo mortality rates than the ZIKV^AS^ isolates (Figure 6A,B). Therefore, even at a lower dose, ZIKV^AF^ is more lethal than ZIKV^AS^ in chicken embryos.

## 4. Discussion

Numerous studies have observed lineage-specific differences in Zika virus epidemiology since the outbreak in the Americas [27,30,32,36,58,59,60]. The results of this study demonstrate that many of these differences are rooted in genetic variation within the viral genome. For instance, the four ZIKV isolates used in this study had similar abilities to grow in basic mammalian cells, but growth varied in C6/36 *Ae. albopictus* cells, and ZIKV^AF^ isolates were more cytopathic to Vero cells. Lineage-specific differences were also observed in vivo; ZIKV^AF^ isolates caused higher mortality in chicken embryos than ZIKV^AS^ isolates, and African-lineage and Asian-lineage dynamics varied in the primary ZIKV vector, *Ae. aegypti* mosquitoes [45].

ZIKV E protein glycosylation may play a role in these ZIKV lineage-specific phenotypic differences, as it does in related flaviviruses. For instance, plaque morphology differences have been noted between West Nile virus isolates, and it was determined that isolates with glycosylated E proteins produced larger plaques, while those lacking E protein glycans produced smaller plaques [35]. Interestingly, the ZIKV^AS^ isolates used in this study have glycosylated E proteins, while the ZIKV^AF^ isolates have deletions that eliminate the glycosylation sites (Figure 1B)*.* Thus, it is possible that differences in ZIKV plaque morphologies could be due to the E protein glycosylation state of the different isolates. Similarly, studies on West Nile [61] and dengue [62] viruses found that the absence of N-linked glycosylation sites in the viral E protein enhanced initial viral infectivity but reduced overall viral production in C6/36 cells. It is possible that the higher growth rates of ZIKV^AF^ early during infection but lower peak titers in C6/36 cells are also a consequence of unglycosylated E proteins.

Our results indicate that isolate SPH is significantly more affected by treatment with IFN-β than the African-lineage isolates in this study. However, a previous study shows that both lineages of ZIKV equally inhibit the IFN-β response through NS5-mediated degradation of STAT2, a signaling molecule in the paracrine IFN-β pathway [28]. Our results also indicate similar relative STAT2 abundance in Vero cells infected with the different isolates. Thus, other viral factors besides NS5-mediated STAT2 degradation must cause the differential responses to IFN-β treatment observed in our study. For instance, data show that subgenomic flavivirus RNA (sfRNA) produced from the 3’ untranslated regions (UTRs) of ZIKV [63] and related flaviviruses [64,65] can also antagonize the interferon response. Sequence alignments of the isolates used in this study reveal several single nucleotide polymorphisms (SNPs) in the 3’UTRs of ZIKV^AS^ versus ZIKV^AF^. However, there are conflicting hypotheses regarding whether these 3’UTR sequence changes would affect sfRNA structure and function [63,66]. Therefore, reverse genetics screens of 3’UTRs, similar to those conducted with Dengue virus [67], will be necessary to make definitive conclusions about the possible role of sfRNA in this isolate-specific ZIKV/IFN-β interaction.

In the *Ae. aegypti* mosquito model, the ability to establish infection in the mosquito midgut is the main barrier to ZIKV reaching the saliva. Our results indicate that ZIKV^AS^ isolates more effectively establish infection in the midgut and, therefore, had significantly higher probabilities of generating infectious mosquitoes than ZIKV^AF^ isolates; mosquitoes that consumed blood mixed with the ZIKV^AS^ Mex 1-44 isolate had the highest probability of becoming infectious. As previously stated, the mosquitoes used in this study were collected in regions of Mexico where the Mex 1-44 isolate was collected. Thus, because Mex 1-44 produced a higher proportion of infectious mosquitoes, our results support previous studies that suggest higher salivary gland infection efficiency occurs when both the mosquito and viral isolate are derived from the same region [37,45]. Despite these correlations, other studies that describe the dynamics between ZIKV lineages in *Aedes* vectors yield contradictory conclusions; some studies have found ZIKV^AF^ to be more successful at establishing infection in the mosquito host than ZIKV^AS^ [68]. In the present study, ZIKV^AF^ isolate MR766 was only able to infect 8 of the 93 mosquitoes that were fed blood that contained MR766. We did not detect any MR766 positive mosquito heads or saliva, while other studies have detected MR766 in more than 30% of *Ae. aegypti* saliva samples [68]. Reasons for such discrepancies are unknown, however multiple hypotheses have been presented, and factors such as blood source, feed method, fresh versus frozen viral stock, and mosquito cohort size/generation could affect overall results [37,68]. Additionally, a previous study found differential *Ae. aegypti* infectivity when the mosquitoes consumed ZIKV isolates that secreted different amounts of NS1 protein, and this may also be a factor contributing to these observed data variations [69]. Overall, this abundance of inconsistent results supports our effort to create lineage-specific standards of ZIKV dynamics in *Ae. aegypti*.

It is proposed that ZIKV^AS^ isolates are more lethal in murine embryo models if they contain S139N substitution in prM [23]. However, the ZIKV^AF^ isolates used in our study do not contain this substitution and retain the S139 in prM (Figure 1B), and therefore this explanation does not account for the increased mortality associated with ZIKV^AF^ infection in chicken embryos. Other factors may contribute to the elevated mortality rate associated specifically with ZIKV^AF^. Studies show that chickens develop an interferon response during embryogenesis, and that the level of IFN-β increases more than 50-fold at later points during development [70]. Chickens lack retinoic acid-inducible gene I (RIG-I), a signaling molecule in the IFN-β pathway that is activated upon flavivirus infection, but have Mda5, a RIG-like molecule that can also lead to IFN-β production [71,72,73]. Our results indicate that ZIKV^AF^ isolates and Asian-lineage Mex 1-44 are affected by human IFN-β, but not to the same extent as SPH. Interestingly, these results correlate with the chicken embryo survival curves; embryos infected with ZIKV^AF^ had the lowest percent survival, followed by those infected with Mex 1-44, and embryos infected with SPH had the highest percent survival (Figure 6A,B). These results may also explain the birth defects associated with congenital ZIKV^AS^ infections that were not reported with ZIKV^AF^ infections; ZIKV^AF^ is more lethal to embryos and, thus, could potentially result in miscarriage, whereas infection with the less lethal ZIKV^AS^ could potentially allow fetuses with ZIKV-induced brain abnormalities to survive. Therefore, an Mda5-induced chicken IFN-β response may play a role in ZIKV virulence in chicken embryos, and the IFN-β response may be similarly important in human gestational ZIKV infections.

Overall, these results provide broad, but informative, lineage-specific ZIKV phenotypes in a variety of laboratory models. However, this study did not define the mechanisms behind these differences; genetic manipulation of ZIKV molecular clones will help determine which genes are responsible for the phenotypic differences observed in this study. Nonetheless, our data show that there are clear differences in ZIKV infection dynamics across these models and, therefore, choice of ZIKV lineage and isolate is essential to experimental design. Ultimately, these results confirm that there are genetic variations that contribute to phenotypic differences between lineages, which are vital for understanding the emergence of Zika virus as a global health threat.

## Figures and Tables

**Table 1:** Zika virus (ZIKV) isolates used in these experiments.

Isolate	Genbank Accession Number	First Reference	Lineage	Isolation Location	Isolate Source	Passage Number in This Study
MR766	LC002520	Dick et al., 1952 [1]	African	Uganda, 1947	Rhesus macaque	>100
IbH 30656	KU963574	Haddow et al., 2012 [12]	African	Nigeria, 1968	Human patient	>25
SPH	KU321639.1	Faria et al., 2016 [15]	Asian	Brazil, 2015	Human patient	<10
Mex 1-44	KX856011.1	Guerbois et al., 2016 [45]	Asian	Mexico, 2015	*Aedes aegypti*	<10

**Table 2:** Results from our mixed effects generalized linear models (binomial distribution; replicate = random factor) and Tukey honest significant difference (HSD) adjusted pair-wise comparisons.

	Probability of Infection	Probability of Dissemination	Probability of Infectiousness	Efficiency of Midgut Escape	Efficiency of Salivary Gland Invasion
ZIKV isolate	F = 34.77 d.f. = 3 *p* < 0.0001	F = 14.71 d.f. = 3 *p* < 0.0001	F = 8.64 d.f. = 3 *p* < 0.0001	F = 6.00 d.f. = 3 *p* = 0.001	F = 1.794 d.f. = 2 *p* = 0.171
Tukey HSD	All pairwise comparisons, *p* < 0.0001	Mex 1-44 vs. SPH, N.S.; all remaining pairwise comparisons, *p* < 0.0001	IbH vs. MR766, N.S.; Mex 1-44 vs. SPH, N.S.; all remaining pairwise comparisons, *p* < 0.0001	Mex 1-44 vs. SPH, N.S.; all remaining pairwise comparisons, *p* < 0.0001	All pairwise comparisons are N.S.

N.S.: not significant; d.f.: degrees of freedom.

**Table 3:** Summary of mosquito infection data.

Isolate	Trial	Blood Meal Titer (log_10_ Plaque Forming Units (PFU)/mL)	Total Blood-Fed (*n*)	Percent Infected Bodies (*n*)	Percent Infected Heads (*n*)	Percent Infected Saliva (*n*)
IbH 30656	1	6.0	48	22.92 (11)	9.09 (1)	0.00 (0)
	2	5.9	31	6.45 (2)	50.00 (1)	0.00 (0)
	3	5.9	39	46.15 (18)	44.44 (8)	50.00 (4)
MR766	1	6.2	46	8.70 (4)	0.00 (0)	0.00 (0)
	2	6.2	47	8.51 (4)	0.00 (0)	0.00 (0)
SPH	1	5.8	36	58.33 (21)	52.38 (11)	18.18 (2)
	2	5.8	37	70.27 (26)	92.73 (24)	45.83 (11)
	3	6.6	43	65.12 (28)	71.43 (20)	35.00 (7)
Mex 1-44	1	6.8	47	93.62 (44)	81.82 (36)	50.00 (18)
	2	6.7	43	62.79 (27)	37.04 (10)	60.00 (6)

Percent infected bodies: proportion of ZIKV-positive bodies to total blood-fed mosquitoes; percent infected heads: proportion of ZIKV-positive heads to total ZIKV-positive bodies; percent infected saliva: proportion of ZIKV-positive saliva to total ZIKV-positive heads.

**Table 4:** ZIKV particles injected per embryonated egg.

Isolate	Infection	Injection Titer (Viral Particles/Egg)
IbH 30656	Low dose	7
	High dose	72
MR766	Low dose	5
	High dose	54
SPH	Low dose	9
	High dose	94
Mex 1-44	Low dose	17
	High dose	168

**Table 5:** Percentage of ZIKV-positive embryos and their average titers.

Isolate	Infection	Percent ZIKV-Positive Embryos	Average Titer (ZIKV-Positive Embryos)
IbH 30656	Low dose	85.71	8.86 × 10^7^
	High dose	94.44	7.58 × 10^8^
MR766	Low dose	69.23	1.42 × 10^8^
	High dose	100	2.92 × 10^9^
SPH	Low dose	22.22	2.49 × 10^8^
	High dose	44.44	9.42 × 10^8^
Mex 1-44	Low dose	30	6.50 × 10^6^
	High dose	88.89	8.78 × 10^7^

**Table 6:** Statistics for chicken embryo survival curves.

Low Titer Injection *p*-Values					High Titer Injection *p*-Values				
	IbH	MR766	SPH	Mex 1-44		IbH	MR766	SPH	Mex 1-44
MR766	0.36				MR766	0.5521			
SPH	0.0194	0.2595			SPH	0.0001	0.0001		
Mex 1-44	0.0339	0.3275	0.778		Mex 1-44	0.0519	0.0245	0.0437	
Sham	0.0001	0.0036	0.0435	0.0215	Sham	0.0001	0.0001	0.0001	0.0001

*p*-values were generated by the logrank (Mantel-Cox) test on Graphpad Prism. The Bonferroni-corrected significance threshold for multiple comparisons is *p* < 0.005.
